# First-in-Human Explantation for Infection of the Novel Thoracic Branched Endograft

**DOI:** 10.1016/j.jaccas.2025.105479

**Published:** 2025-10-29

**Authors:** Fabian Jimenez Contreras, Griffin P. Stinson, Patrick D. Kohtz, Brian F. Gilmore, Gilbert R. Upchurch, Tomas D. Martin

**Affiliations:** a Division of Thoracic and Cardiovascular Surgery, Department of Surgery, University of Florida, Gainesville, Florida, USA; b Department of Surgery, Division of Vascular Surgery, University of Florida, Gainesville, Florida, USA

**Keywords:** endograft explantation, thoracic branched endograft, mycotic aneurysm

## Abstract

**Background:**

The Gore TAG thoracic branched endoprosthesis (W.L. Gore & Associates) is the only branched thoracic stent graft available in the U.S.

**First-in-Human/Early Reports Summary:**

A 67-year-old man underwent thoracic branched endograft (TBE) placement with the Gore TAG device for a contained rupture of the distal aortic arch and thoracic aorta. Imaging showed a mycotic aneurysm that eroded into the left lower lobe, causing hemoptysis. He was transferred to a quaternary care aortic center.

**Discussion:**

To remove the TBE, a left common carotid–to–subclavian artery bypass was performed. The patient was cannulated and placed in hypothermic circulatory arrest. The aneurysm was explored, and both stent components were explanted. The aorta was reconstructed with anastomoses in zones 2 and 5. Two additional washouts were required, but the patient had an uncomplicated course and was discharged on postoperative day 18.

**Novelty:**

To our knowledge, this is the first reported case of explantation of an infected TBE.

**Take-Home Message:**

Explantation of an infected TBE is feasible with multidisciplinary care at an experienced aortic center.

## Graphical Abstract

**Figure:**
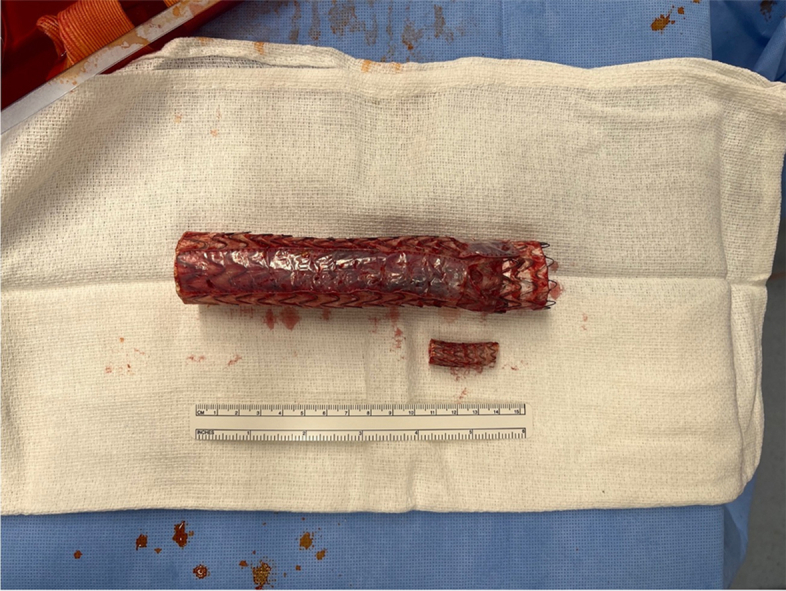

## Background

Thoracic branched endografts (TBEs) allow for endovascular management of proximal thoracic aortic disease in patients unfit for open repair. Though rare, endograft infection can occur in 1.5% to 2% of interventions.^1^^,^^2^ The Gore TAG thoracic branched endoprosthesis (W.L. Gore & Associates) received U.S. Food & Drug Administration approval in May 2023 and remains the only branched thoracic stent graft approved for use in the United States. As TBE use expands, management of complications will become increasingly important. Herein, we present to our knowledge the first reported case of TBE explantation for infection.

**Take-Home Messages**

• Explantation of an infected thoracic branched endograft required collaboration between cardiac surgery, vascular surgery, acute care surgery, critical care medicine, and infectious disease medicine.
• Long-term outcomes, freedom from subsequent infection, and the need for additional intervention are unclear given the rarity of this procedure and require further experience.

## First-in-Human Summary

A 67-year-old man underwent TBE placement with left subclavian artery extension at another institution for a zone 3 contained rupture of a pseudoaneurysm of unknown origin. At the time of presentation, the patient did not show any clinical signs of infection, and given the emergent nature of the procedure, blood cultures were not drawn. Thus, pre-existing infection cannot be ruled out. He was discharged on postoperative day 5 without complication but returned 8 days later with chest pain. Computed tomography angiography (CTA) showed a zone 3 pseudoaneurysm with enlarging, mildly hyperdense fluid collection in the proximal descending thoracic aorta and a type 1a endoleak. The TBE was extended approximately 2 cm proximally using the Gore TBE proximal extension component to terminate 6 mm distal to the left common carotid artery on the outer curvature. Two days after this reintervention, CTA suggested a mycotic aneurysm. The patient's outside hospital course was further complicated by persistent hemoptysis and pulseless electrical activity cardiac arrest with resuscitation. A flexible bronchoscopy was performed, demonstrating a bleeding site in the left upper lobe apicoposterior segment that was sealed with fibrin sealant. Given such significant bleeding and concern for aortobronchial fistula, the patient underwent open thoracotomy with left upper lobe wedge resection and Dacron graft buttress of aorta. He was then transferred to our institution for definitive surgical management of his suspected persistent mycotic aneurysm.

**Figure 1:**
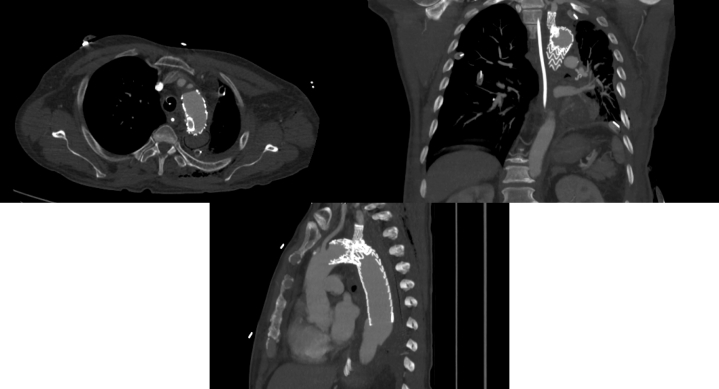
Preoperative Computed Tomography Scans of a Large Perigraft Fluid/Gas Collection

CTA upon transfer demonstrated a large (18-cm craniocaudal) perigraft fluid/gas collection contiguous with the left posterior pleura, as well as a 4.0-cm saccular aneurysm of the noncovered portions of zones 4/5 (Figure 1). Removal of his infected endograft and replacement of the arch and descending aorta was planned. The patient was brought to the operating room and placed supine for a left carotid–to–subclavian artery bypass with an 8-mm Dacron graft, which was completed without complication.

The patient was repositioned in the right lateral decubitus position for the subsequent thoracic intervention. The left femoral vessels were exposed and cannulated for cardiopulmonary bypass (CPB). CPB was initiated, and the patient began cooling to 18 °C. The previous thoracotomy incision was simultaneously extended caudally and was used to access the left chest. Once cooled, the patient was placed into the Trendelenburg position, circulatory arrest was achieved, and the aorta was clamped distally in zone 5 to allow partial CPB to the lower body and halt perfusion of the arch.

**Figure 2:**
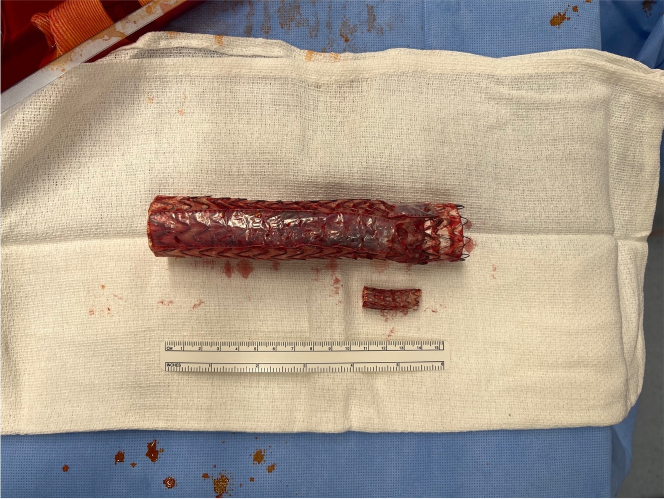
Explanted Thoracic Branched Endograft With Left Subclavian Stent

The TBE was exposed proximally and distally. There was significant necrosis and infected tissue. The left phrenic nerve was able to be identified and preserved. The left vagus nerve was completely encased in fibrosis and was unable to be preserved. To prevent debris embolization, a surgical sponge was placed into the ascending aorta. There was infected aortic tissue in zones 2 to 4, similar to the area of the original contained rupture. The left subclavian artery stent was cut, and the TBE was easily removed. The subclavian stent fell from the subclavian artery, and a large abscess involving the proximal left subclavian artery was revealed. The totality of the previous TBE and extension were removed (Figure 2).

The aorta was transected at the lesser curvature of the aortic arch with a long bevel up to the orifice of the left subclavian artery. Nearly the entire aortic arch was reconstructed with a 28-mm rifampin-soaked side-arm Dacron graft, with anastomoses along the lesser curvature bevel at zone 0 and up into the orifice of the left common carotid artery, even with the innominate artery, in what can be described as a reverse hemiarch. All aortic anastomoses were completed in standard running fashion with 3-0 polypropylene, and then Bioglue was applied. The distal graft was clamped, and utilizing the side arm, CPB was used to perfuse the heart, innominate artery, and left common carotid arteries. Circulatory arrest time was 33 minutes.

The proximal left subclavian artery was then ligated. Significant necrosis involving zones 3 and 4 was noted. The graft was then anastomosed distally onto zone 5 and de-aired. CPB was increased to full flow, and full body perfusion was re-established, A gentamycin irrigation catheter was placed on top of the Dacron graft. The patient was decannulated, with total CPB time at 196 minutes. He was taken to the cardiac intensive care unit in stable condition. Cultures of intraoperative swabs of both the descending thoracic aneurysm cavity and of the aortic endograft grew methicillin-resistant *Staphylococcus aureus* (MRSA).

Owing to the extent of infection and necrosis involving the endograft, the patient required multiple subsequent chest washouts as an anticipatory measure. On postoperative day 3, he underwent a planned return to the operating room for washout to prevent infection propagation. On postoperative day 6, he underwent a final washout with omental flap coverage of the graft by acute care surgery and pericardial window creation for persistent, noninfectious pericardial effusion. He was later transitioned to the floor on postoperative day 10. The patient was discharged home on postoperative day 18 with a 6-week course of vancomycin that was switched to daptomycin given concern for leukocytoclastic vasculitis, and lifelong suppressive doxycycline after consultation with the infectious disease medicine team.

## Discussion

Infection of aortic endografts is a devastating complication and requires open reintervention to remove the infected graft, necrotic portions of the aorta, and repair of aortic defects.^1^^,^^2^ This case is to our knowledge the first reported explantation of a TBE, but it will not be the last as TBE use increases. The infected graft was first evaluated using CTA, as would any aortic disease. Along with CTA, bronchoscopy was useful for the identification and temporization of an aortobronchial fistula before wedge resection. Through a rare complication, aortobronchial fistulas can occur in the setting of infected aortic hardware in which the organism, particularly those that are Staphylococcal, erodes into the adjacent bronchial tree.^3^ These fistulas are typically heralded by hemoptysis, as was seen here.^4^ Other imaging we used included transesophageal echocardiography, though this was primarily for preoperative cardiac evaluation rather than surgical planning.

The infected graft was then accessed through a left lateral thoracotomy, rather than median sternotomy. In this case, a left lateral approach allowed for direct access to the infection and totality of the proximal and distal extents of the TBE. As there was no intracardiac infection or abscess, a median sternotomy was not required, though it can be considered for patients with aortic root or valvular involvement. The totality of the TBE was excised with all known infected/necrotic material to mitigate infection recurrence. Though the infected graft included subclavian stenting and necrosis, the management did not deviate far from nonbranched infected endograft treatment, with the addition of resection and ligation of the infected left subclavian artery.^1^^,^^2^^,^^5^ Placement of a gentamycin irrigation catheter allowed for the postoperative irrigation of the reconstructed graft with the chest remaining closed. This is now a routine practice at our center, as we believe it reduces postoperative infection propagation in infected operative fields.

After reintervention, an omental flap was used to cover the newly implanted graft, as reinfection was suspected to occur.^3^ Though it requires reopening of both the chest and abdomen, omental flap coverage allows for adequate blood supply, systemic antibiotic delivery, and lymphatic drainage to be overlaid onto the newly implanted graft and infected surrounding tissue.^6^^,^^7^ This has been demonstrated as a powerful adjunct to combat reinfection after infected graft explantation.^6^, ^7^, ^8^ Major complications and additional infections have been avoided because of diligent thoracic washouts and debridement. For the patient's antibiotic management, a 6-week course of vancomycin and subsequently daptomycin were chosen, as his infection and surrounding necrosis were easily visible upon surgical visualization.^2^ Further, lifelong suppressive doxycycline was chosen because the patient had a significant MRSA infection, had a history of previous aortic interventions, had extensive perigraft infection and fistulization, and was a poor surgical candidate for additional reoperation.^2^ The total duration of therapy with rifampin, vancomycin/daptomycin, and suppressive doxycycline was confirmed with the infectious disease medicine team.

## Novelty

Surgical management of infected TBE may be more complicated than management of nonbranched endograft infections, requiring extensive debridement and reconstruction under circulatory arrest, as seen here. This initial report suggests that treatment of TBE infection can be successful if performed at a large-volume aortic center.

## Funding Support and Author Disclosures

The authors have reported that they have no relationships relevant to the contents of this paper to disclose.

## References

[1] O'Connor S., Andrew P., Batt M., Becquemin J.P. (2006). A systematic review and meta-analysis of treatments for aortic graft infection. J Vasc Surg.

[2] Wilson W.R., Bower T.C., Creager M.A. (2016). Vascular graft infections, mycotic aneurysms, and endovascular infections: a scientific statement from the American heart Association. Circulation.

[3] Fitridge R., Thompson M. (2011). Mechanisms of Vascular Disease: A Reference Book for Vascular Specialists.

[4] Peacock T., Limmer A.M., Zahid A. (2023). Aorto-bronchial fistula masquerading as haematemesis: a rare late complication of thoracic aortic vascular grafts. J Surg Case Rep.

[5] Kan C.D., Lee H.L., Yang Y.J. (2007). Outcome after endovascular stent graft treatment for mycotic aortic aneurysm: a systematic review. J Vasc Surg.

[6] Hernandez J.A., Stranix J.T., Piwnica-Worms W. (2020). Omental flap coverage for management of thoracic aortic graft infection. Ann Thorac Surg.

[7] Andrade D., Vinck E.E., Torres L.N. (2020). Two-stage omental flap approach for ascending aortic graft infection. Braz J Cardiovasc Surg.

[8] Tomotsuka S., Takehara M., Tsumaru S., Shimamoto T. (2023). A novel technique to wrap, not merely cover, the omental flap around the infected graft. Ann Thorac Surg Short Rep.

